# Analytical and clinical validation of a blood progranulin ELISA in frontotemporal dementias

**DOI:** 10.1515/cclm-2023-0562

**Published:** 2023-07-24

**Authors:** Francisco Meda, Joel Simrén, Barbara Borroni, Valentina Cantoni, Silvana Archetti, Giorgio Biasiotto, Ulf Andreasson, Kaj Blennow, Hlin Kvartsberg, Henrik Zetterberg

**Affiliations:** Department of Psychiatry and Neurochemistry, Institute of Neuroscience and Physiology, The Sahlgrenska Academy at the University of Gothenburg, Mölndal, Sweden; Clinical Neurochemistry Laboratory, Sahlgrenska University Hospital, Mölndal, Sweden; Centre for Neurodegenerative Disorders, Department of Clinical and Experimental Sciences, University of Brescia, Brescia, Italy; Department of Molecular and Translational Medicine, University of Brescia, Brescia, Italy; Department of Neurodegenerative Disease, Dementia Research Centre, UCL Institute of Neurology, Queen Square, London, UK; UK Dementia Research Institute at UCL, London, UK; Hong Kong Center for Neurodegenerative Diseases, HKCeND, Hong Kong, China; Wisconsin Alzheimer’s Disease Research Center, University of Wisconsin School of Medicine and Public Health, University of Wisconsin-Madison, Madison, WI, USA

**Keywords:** frontotemporal dementia, progranulin, validation

## Abstract

**Objectives:**

Heterozygous mutations in the granulin (*GRN*) gene may result in haploinsufficiency of progranulin (PGRN), which might lead to frontotemporal dementia (FTD). In this study, we aimed to perform analytical and clinical validation of a commercial progranulin kit for clinical use.

**Methods:**

Analytical validation parameters including assay precision, selectivity, measurement range, dilution linearity, interferences and sample stability were tested according to previously described procedures. For clinical validation, PGRN levels were measured in plasma from 32 cognitively healthy individuals, 52 confirmed *GRN* mutation carriers, 25 *C9orf72* mutation carriers and 216 patients with different neurodegenerative diseases of which 70 were confirmed as non-mutation carriers.

**Results:**

Among the analytical validation parameters, assay precision and repeatability were very stable (coefficients of variation <7 %). Spike recovery was 96 %, the measurement range was 6.25–400 μg/L and dilution linearity ranged from 1:50–1:200. Hemolysis did not interfere with progranulin levels, and these were resistant to freeze/thaw cycles and storage at different temperatures. For the clinical validation, the assay was capable of distinguishing *GRN* mutation carriers from controls and non-*GRN* mutation carriers with very good sensitivity and specificity at a cut-off of 57 μg/L (97 %, 100 %, respectively).

**Conclusions:**

In this study, we demonstrate robust analytical and diagnostic performance of this commercial progranulin kit for implementation in clinical laboratory practice. This easy-to-use test allows identification of potential *GRN* mutation carriers, which may guide further evaluation of the patient. This assay might also be used to evaluate the effect of novel PGRN-targeting drugs and therapies.

## Introduction

Frontotemporal dementia (FTD) is a diverse neurodegenerative disease with distinct phenotypes. It mainly affects the frontal and temporal lobes of the brain resulting in behavior, personality, or language deficits [1]. It is considered the second most common form of early onset dementia, after Alzheimer’s disease (AD) and presents itself in several subtypes. The behavioral variant frontotemporal dementia (bvFTD) is characterized by disinhibited and repetitive behavior, irritability and reduced empathy, while primary progressive aphasia (PPA) subdivides into 3 subtypes: semantic variant (svPPA), non-fluent variant (nfvPPA), and logopenic variant (lvPPA); all of which are related to agrammatism and apraxia of speech [2, 3]. Motor deficits can also be coupled with FTD, namely amyotrophic lateral sclerosis (FTD-ALS), or parkinsonism with elements of progressive supranuclear palsy (PSP) and corticobasal syndrome (CBS) [4].

The primary hallmarks of FTD include protein aggregates, neurodegeneration, and lysosomal dysfunction, with the most common protein aggregates being TDP-43 (<50 %), tau (∼45 %) and other proteins (5–10 %) [5]. FTD can be either sporadic or familial. For familial FTD, it is hereditarily transmitted as autosomal dominant with nearly 100 % penetrance, almost guaranteeing the development of the disease. These mutations are derived from three specific genes: progranulin (*GRN*), chromosome 9 open reading frame 72 (*C9orf72*) and microtubule-associated protein tau (*MAPT*). *C9orf72* mutations are the most common cause of FTD worldwide, followed by *GRN* and *MAPT* with 114 and 63 mutations identified respectively [4]. More rarely, other genes might be involved in FTD pathophysiology, including *TBK1*, *SQSTM1*, *TARDBP*, *VCP*, and *CHIM2B* [6].

The *GRN* gene is located on chromosome 17q21 and encodes for the 88 kDa glycoprotein progranulin (PGRN). This protein comprises 7.5 tandem repeats of 12 cysteine motifs separated by spacer regions and is processed by cathepsins into seven 6 kDa granulins in the lysosome [7, 8]. PGRN is expressed in a variety of tissues and is involved in development, wound repair, and inflammation [9]. It may act as growth factor, anti-inflammatory agent or adipokine, depending on the target tissue. In the brain, it is present in activated microglia and neurons, and regulates neurite branching and neuroinflammation [10], [11], [12]. While PGRN has anti-inflammatory properties (through competition with TNF-α for receptor-binding [13, 14]), its cleavage products, the granulins, work the opposite way, with pro-inflammatory actions [15].

While heterozygous *GRN* mutations may lead to FTD development, homozygous mutations cause neuronal ceroid lipofuscinosis, a lysosomal storage disease characterized by lipofuscin accumulation [16]. Besides that, aberrant PGRN expression is also involved in some autoimmune diseases and has been studied in tumorigenesis [14, 17].

Most of these mutations are either frameshift or, more often, nonsense, creating null alleles by introducing a premature stop codon. This consequently reduces PGRN expression ∼50 % and leads to FTD due to PGRN haploinsufficiency [18, 19]. PRGN alterations were also proven to influence disease progression, with lower expression contributing to faster disease progression [20]. This deficiency is so significant that, in plasma, PGRN expression distinguishes *GRN* mutation carriers from controls and non-*GRN* mutations carriers with very good sensitivity and specificity [20], [21], [22]. However, plasma PGRN levels correlate poorly with cerebrospinal fluid (CSF) PGRN [23, 24]. Besides that, various single nucleotide polymorphisms (SNP) have been associated with altered levels of PGRN: rs5848 (*GRN*), rs646776 in the sortilin 1 gene (*SORT1*), rs1990622 in *TMEM106B*, rs1867977 in prosaposin (*PSAP*), as well as in the cadherin 23 (*CDH23*) locus. These SNPs showed altered PGRN expression dependent on the number of allele copies [8, 25]. Minor allele rs5848 also negatively impacts survival after onset in *C9orf72* mutation carriers [26].

*TMEM106B*, in particular, is considered as a risk factor for FTD-*GRN*. It impacts lysosome acidification, enlargement, disrupting trafficking and function. Its overexpression decreases production of granulins and PGRN secretion, increasing intracellular PGRN [13, 27]. However, *TMEM106B* minor alleles rs1990622 and rs6966915 correlated with increased PGRN levels [25].

Although several assays are available for PGRN quantification, all have different measurement ranges and produce inconsistent results between one another. In this study, we aimed to perform analytical and clinical validation of one commercial progranulin kit in the FTD spectrum disorders for clinical use of this biomarker. Here, we prove the robustness and precise diagnostic performance of this kit, which accurately distinguishes *GRN* mutations carriers from other neurodegenerative disease patients and non-*GRN* mutation carriers. This allows for a straightforward screening of patients to identify individuals who are *GRN* mutation carriers, which may improve the diagnostic accuracy.

## Materials and methods

### Progranulin ELISA protocol

Samples were analyzed in duplicate, randomized, and blinded to the mutation or disease type. PGRN concentrations in plasma or serum were measured using the established enzyme-linked immunosorbent assay kit from Adipogen [Progranulin (human) ELISA Kit, AdipoGen Life sciences, cat#: AG-45A-0018YTP-KI01] following the manufacturer instructions, apart from sample dilution which was adjusted to 1/100 to ensure that all measured concentrations fell within the range of the calibration curve. Low and high concentration quality controls (LQC, HQC) were used on each plate and an internal calibrator (IC) was also included to normalize absolute values between plates.

### Collection process correlation

Whole blood from 10 anonymized patients was collected in ethylenediaminetetraacetic acid (EDTA) tubes or lithium heparin tubes for plasma, and regular collection tubes for serum extraction. After centrifugation at 2000 *g* for 10 min, plasma and serum were aliquoted and stored at −80 °C until analysis. The three collected sample types were then analyzed in duplicate, and correlations between sample types were assessed.

### *GRN *genetic screening

Genomic DNA was obtained from 400 µL of EDTA-anticoagulated peripheral blood, according to standard procedures. *GRN* gene sequencing was carried out using the Ion Torrent PGM platform (Thermo Fisher Scientific, Waltham, MA USA) by the Ion PGM Hi-Q Sequencing Kit (Thermo Fisher Scientific, Waltham, MA USA). Genetic variants were assessed by Ion Reporter 5.2 Software (Thermo Fisher Scientific, Waltham, MA USA), further verified using Integrative Genomic Viewer (IGV software, Broad Institute, University of California, USA). All pathogenetic variations were confirmed by Sanger Sequencing (SeqStudio, Applied Biosystem by Thermo Fisher Scientific, Waltham, MA USA).

### Participants

This retrospective study included participants from the Centre for Neurodegenerative Disorders, University of Brescia, Italy, fulfilling clinical criteria of bvFTD [2], PPA [3], CBS [28], PSP [29], and AD [30]. Moreover, subjects at risk to develop FTD because carriers of pathogenetic mutations and healthy controls (HC) were enrolled as well.

Each patient underwent a neurological evaluation, routine laboratory examination and a neuropsychological and behavioural assessment. In all cases the diagnosis was supported by brain structural imaging, while cerebrospinal fluid concentrations of tau, p-Tau181 and Aβ1-42 were measured in a subset of cases to rule out AD, as previously reported [31]. Furthermore, genetic screening for *GRN* and *C9orf72* was carried out according to standard procedures [31].

This study was composed of 64 AD (age, mean ± SD, 69.6 ± 7.8, female%=54.7), 94 bvFTD (age, 64.2 ± 13.8, female%=34.0), 65 PPA (age, 65.9 ± 8.2, female%=66.2), 26 CBS (age, 68.9 ± 6.5, female%=46.2), 18 PSP (age, 72.6 ± 13.7, female%=55.6). Among these patients, 31 subjects were *GRN* mutation carriers and 20 subjects were *C9orf72* expansion carriers. Moreover, 21 pre-symptomatic *GRN* mutation carriers (age, 48.2 ± 10.9, female%=61.9), 5 pre-symptomatic *C9orf72* expansion carriers (age, 43.4 ± 13.2, female%=20.0), and 32 HC (age, 46.7 ± 17.2, female%=65.6) were included.

Full written informed consent was obtained from all subjects according to the Declaration of Helsinki. The Brescia Ethics Committee approved the study protocol.

### Assay validation

#### Repeatability and intermediate precision

Plasma from anonymized patients was pooled into 3 separate batches. The first pool (90 μg/L) was diluted 1:3 with PBS 1 % BSA to about 30 μg/L and was used as a low QC. The second pool (130 μg/L) was used as high QC and the third pool (75 μg/L) was used as IC. IC was used for normalization, to compensate for variations between plates and kit batches. Pools were aliquoted and stored in cryotubes at −80 °C until measurement. The samples were analyzed in duplicates five times on five different occasions, with two batches of kits and by two different lab technicians. The IC was also analyzed in duplicate in the five different occasions. In this particular case, as progranulin levels between groups are quite distinct, repeatability and intermediate precision were accepted if the coefficients of variation (CV) were below 15 %.

#### Linearity test/spike recovery

To test dilution linearity, five plasma samples with concentration between 84 and 164 μg/L were serial diluted 50, 100 and 200 times in analysis buffer. Values within the 11 % variation limit were accepted. This measurement uncertainty was used to assess further results and based on the highest measured repeatability (5.6 %) with a coverage factor of 2. Spike recovery was assessed using five individual plasma samples. These were diluted, according to the assay protocol, and divided into two aliquots each, one of which was spiked with a small volume of progranulin calibrator with the ratio of spike to sample volume being 1:20 (1 part progranulin calibrator, 19 parts plasma). The same volume of analysis buffer was added to the second aliquot. All samples were analyzed in duplicate in the same analytical run.

#### Measurement range

Data from the calibration curves, utilizing 5-parametric logistic regression with 1/Y^2^ weighting, from five experiments performed with two batches of kits, by two laboratory technicians, were used and residuals calculated for all points. The measurement range was defined at the interval of the calibration curve for which all calibrator points had a relative <11 % deviation from the nominal concentration.

#### Interferences and hook effect

As the matrix of choice for this assay was plasma, hemolysis interference was also assessed. Whole blood from 6 individuals was collected in EDTA tubes. From each individual, one EDTA tube was centrifuged at 2000 *g* for 10 min. Plasma was collected and aliquoted four times, 150 µL/tube and stored at −80 °C until further analysis. The other EDTA tube was frozen without centrifugation to induce hemolysis. Upon analysis, hemolyzed samples were centrifuged at 4000 *g* for 10 min. Out of the four aliquots, two were spiked with 3 µL of matched hemolyzed sample and the other two were left untouched. All tubes were centrifuged at 2000 *g* for 10 min at room temperature before analysis.

#### Sample stability

**Figure 1:**
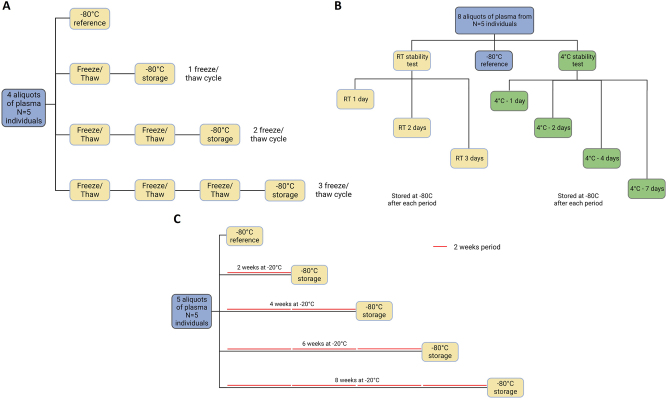
Workflow of sample handling for different validation steps. (A) Sample stability to freeze/thaw cycles schematic. Four aliquots from five plasma samples were tested from reference (placed straight into −80 °C) up to 3 freeze/thaw cycles. (B) Room temperature and fridge (4 °C) sample stability test schematic. From five plasma samples, three aliquots were left, one, two and three days at RT, one aliquot immediately frozen at −80 °C used as reference and four aliquots left in the fridge for one, two, four and seven days. Every sample was stored at −80 °C after each period. (C) Long term freezer (−20 °C) stability test schematic. Four aliquots from five plasma samples were kept in the freezer for two, four, six and eight weeks, while one was kept at −80 °C for reference. RT, room temperature.

Stability was tested in four different ways: Freeze/thaw cycles, room temperature, cold stability and long-term freezer stability. All samples used were from anonymized individuals. Freeze/thaw stability was assessed with four aliquots of plasma from five different individuals, stored at −20 °C. One aliquot did not undergo any freeze/thaw cycles, while the other three underwent 1–3 cycles, respectively. Each freeze/thaw cycle was performed on the bench at room temperature for 1.5 h, vortexed, lid opened and put back on the freezer until the next freeze/thaw occasion. When the required number of cycles was achieved the samples were stored at −80 °C until analysis (Figure 1A).

Room temperature and cold stability were determined using 5 plasma samples aliquoted 8 times with one of the aliquots stored immediately at −80 °C, serving as reference. For room temperature, three aliquots were left on a bench exposed to daylight for one, two and three days respectively. For cold stability, four aliquots were stored at 4 °C for one, two, four and seven days, respectively. After each corresponding storing period, every sample was kept at −80 °C until further analysis (Figure 1B).

Usually, 8 weeks is the period in which samples in clinical routine are kept before destruction or anonymization. As such, the long-term freezer stability was determined with five plasma samples divided into five aliquots each, up to 8 weeks. One aliquot was immediately stored at −80 °C to serve as reference, while the others were kept in the freezer. Every two weeks one aliquot from each individual was moved to −80 °C, where it was kept until further analysis (Figure 1C).

### Statistical analysis

Non-parametric Kruskal–Wallis test with Dunn´s correction used for all group comparisons and non-parametric Mann–Whitney test used for *GRN* mutation carriers vs. non mutation carriers comparison. ROC analysis was used to assess specificity and sensitivity between *GRN* mutation carriers and non-mutation carriers. Spearman correlation and simple linear regression were used to evaluate differences between collection process. All analysis were performed using GraphPad software version 9.5.1.

## Results

### Repeatability and intermediate precision

A summary of the precision experiments is presented in Table 1. The precision results remained within the pre-determined limit of 15 % and normalization against the IC resulted in higher precision, with the concentration of the IC being 73 μg/L. Furthermore, assay precision slightly improved with subsequent samples tested after this study, with normalization to IC still resulting in lower CVs (4.8 and 6.9 %).

**Table 1:** Repeatability and intermediate precision of PRGN detected in plasma by the Adipogen Life Sciences progranulin kit.

Sample dilution…1:100		Mean concentration, μg/L	SDr, μg/L	CVr, %	SDrw, μg/L	CVrw, %
Analytical report	Low control	32	1.8	5.6	2.2	6.8
	Low control normalized to IC	31	1.7	5.5	1.8	6
	High control	133	6.5	4.9	13	10
	High control normalized to IC	128	6.2	4.8	11	8.5
Cohort and clinical routine	Low QC	34	1.4	4.1	2.5	7.3
	Low QC normalized to IC	32	1.3	4.1	2.1	6.5
	High QC	139	6.7	4.8	9.6	6.9
	High QC normalized to IC	128	6.0	4.7	8.2	6.4

SD, standard deviation; r, repetability; rw, intermediate precision; IC, internal calibrator; CV, coefficient of variation; QC, quality control.

### Dilution linearity and spike recovery

**Figure 2:**
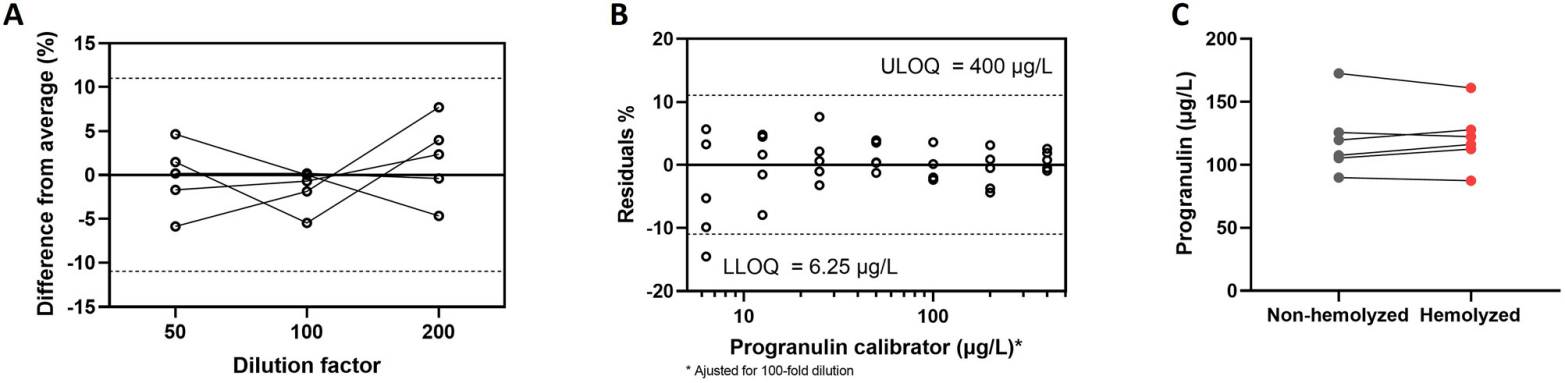
Analytical validation parameters. (A) Dilution linearity from 50 to 200-fold as percentage difference from average, (B) measurement range as residuals percentage with selected LLOQ of 6.25 μg/L and ULOQ of 400 μg/L and (C) hemolysis interference, p=0.82 by non-parametric Mann–Whitney test. Dotted lines indicate 11 % measurement uncertainty. LLOQ, lower limit of quantification; ULOQ, upper limit of quantification.

From the five samples tested, dilution ranging from 50- to 200-fold did not influence the outcome results with no samples going outside 11 % difference from average (Figure 2A).

Recovery results from five samples tested ranged from 88-101 %, averaging at 96 %, which was within the pre-determined limit of 15 % (Table 2).

**Table 2:** Spike recovery results.

Sample	Spike concentration, μg/L	Sample concentration, μg/L	Spiked sample concentration, μg/L	Recovery, %
1	86.0	90.5	175.5	98.9
2	170.0	214.2	385.4	100.7
3	66.0	88.2	154.5	100.5
4	135.0	126.3	249.5	91.3
5	109.0	117.3	212.9	87.6

### Measurement range

The endpoints of the calibration curve define the technical measurement range of this assay. In this case, the lower limit of quantification (LLOQ) is defined at 0.0625 μg/L and the upper limit of quantification (ULOQ) at 4 μg/L, when considering the 100-fold dilution becomes 6.25–400 μg/L (Figure 2B), that goes in accordance with the kit manufacturer’s guidelines.

### Interferences

The degree of hemolysis used for this experiment did not show statistically significant differences between the measured progranulin concentrations (p=0.82, Figure 2C), speaking against hemolysis affecting progranulin concentrations.

### Sample stability

**Figure 3:**
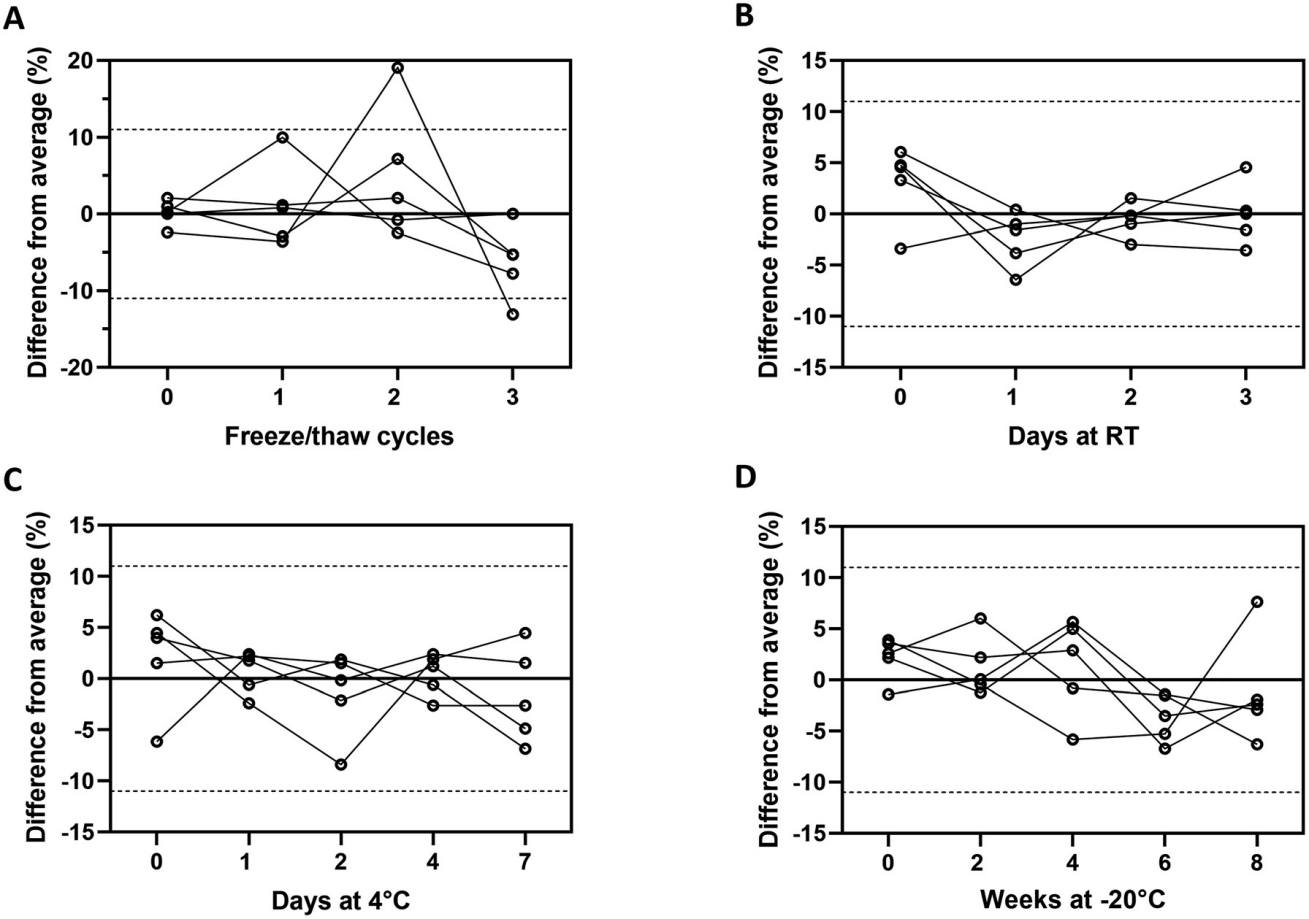
Sample stability at different temperatures. (A) Freeze/thaw cycles endurance, (B) days stored at room temperature, (C) fridge stability and (D) freezer stability, all data is plotted against percentage difference from average. Dotted lines indicate 11 % measurement uncertainty.

According to the experiments performed, PGRN is stable and resistant to freeze-thaw cycles, as well as storage at different temperatures, as all measured values, except one, lie within the established measurement uncertainty (11 %) (Figure 3A). PGRN concentration is also constant if the samples are left at room temperature for up to three days (Figure 3B), at 4 °C up to seven days (Figure 3C) and can also withstand long periods at −20 °C (Figure 3D), with all difference values being within the measurement uncertainty.

### Clinical validation

**Figure 4:**
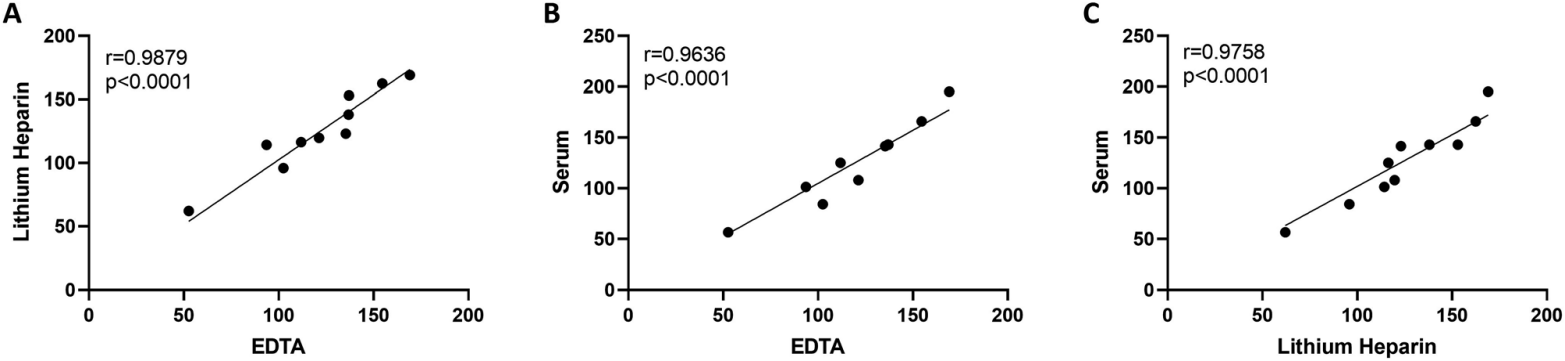
Collection process correlations between plasma (with different anti-clotting agents, EDTA and LiHep), and serum. High correlations shown through all comparisons. (A) LiHep vs. EDTA, r=0.9879, p<0.0001; (B) serum vs. EDTA, r=0.9636, p<0.0001; (C) serum vs. LiHep, r=0.9758, p<0.0001, by Spearman correlation. EDTA, ethylenediaminetetraacetic acid.

After the technical validation of the assay, collection process interference was determined by assessing correlations between collection processes. Comparisons show very high correlation (LiHep vs. EDTA, r=0.9879, p<0.0001; serum vs. EDTA, r=0.9636, p<0.0001; Serum vs. LiHep, r=0.9758, p<0.0001). Besides that, slopes calculated by linear regression (LiHep vs. EDTA, 1.025; serum vs. EDTA, 1.048; Serum vs. LiHep, 1.019), show the interchangeability of these sample types (Figure 4A–C).

**Figure 5:**
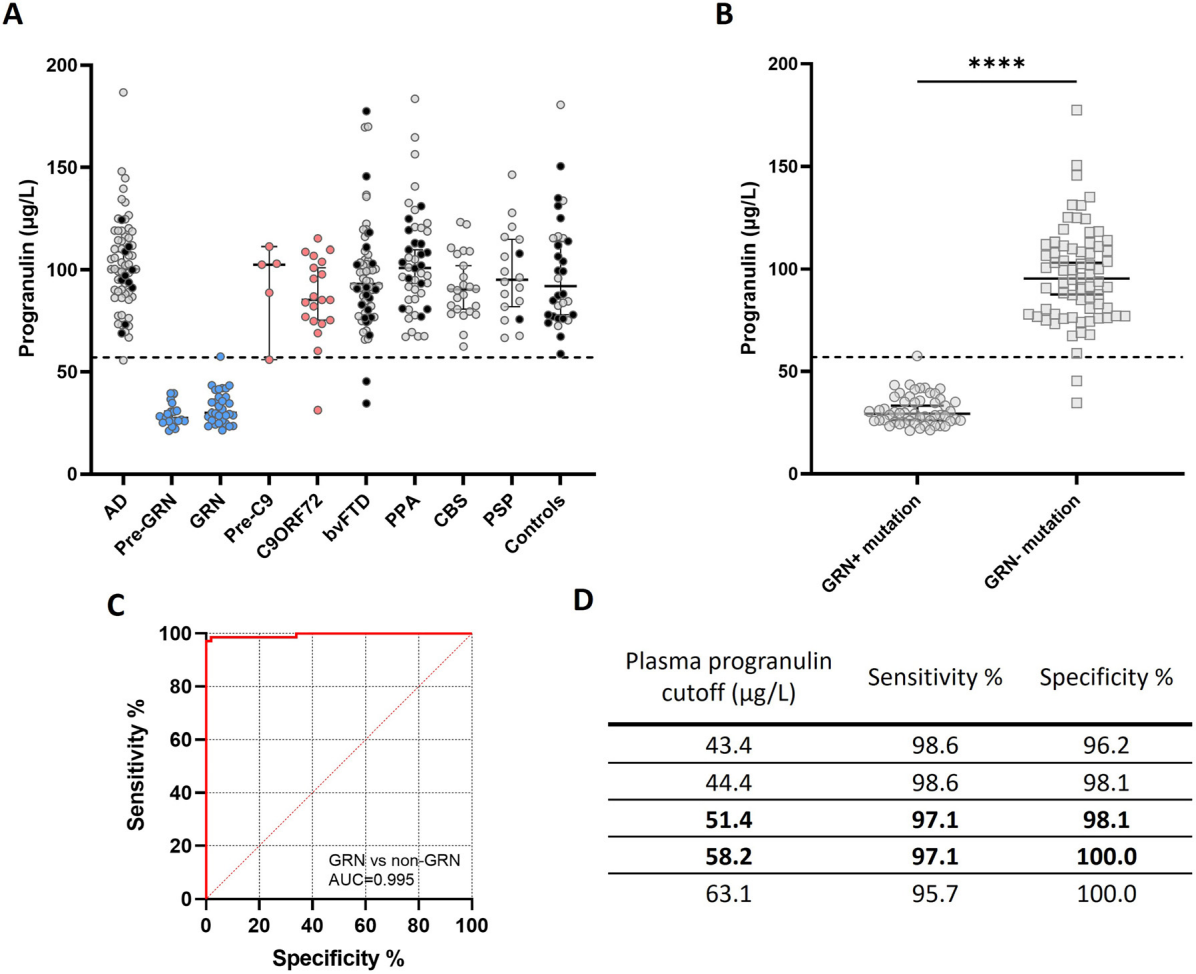
Progranulin concentration in µg/L measured using the Adipogen Life sciences progranulin kit (A) in plasma samples from several neurodegenerative diseases, *C9or72*, *GRN* mutation carriers and healthy controls. Red dots represent confirmed *C9orf72* mutation carriers, blue dots represent *GRN* confirmed mutation carriers and black dots represent confirmed non-mutation carriers. (B) Confirmed *GRN* mutation and non-mutation carriers comparison. *GRN*, granulin; ****p<0.0001 by non-parametric Mann–Whitney test; error bars represent 95 % confidence intervals. Dotted line represents established cutoff at 57 μg/L. ROC analysis of plasma progranulin levels, (C) area under the curve (AUC) of 0.995 provides high discrimination between mutation carriers and non-mutation carriers (p<0.0001), (D) and best cutoffs in bold with respective sensitivity/specificity percentages. Established cutoff of 57 μg/L gave the best overall sensitivity/specificity combination. AD, Alzheimer’s disease; *GRN*, granulin; *C9orf72*, chromosome 9 open reading frame 72; bvFTD, behavioral variant frontotemporal dementia; PPA, primary progressive aphasia; CBS, corticobasal syndrome; PSP, progressive supranuclear palsy; ROC, receiver operation characteristics.

The diagnostic performance was accessed in plasma of individuals with several neurodegenerative diseases (see methods on participants). It is important to mention that in Figure 5A, non-mutation carriers are not distinguished and are spread among all groups. Considering the spectrum of diseases, it is clear that the assay significantly distinguishes *GRN* mutation carriers, either symptomatic or pre-symptomatic, from every other disease type (p<0.0001 for *GRN* and Pre-*GRN* vs. AD, bvFTD, PPA, CBS, PSP and Controls; p<0.001 vs. *C9orf72* mutation carriers; p<0.05 vs. pre-*C9orf72*). Furthermore, there are no other statistically significant differences specially between the pre-symptomatic and symptomatic *GRN* mutation carriers (p>0.999) (Figure 5A).

Including only the confirmed *GRN* mutation and non-mutation carriers by genetic testing, there was a significant difference between the two groups (*GRN* mutation carriers, 31.2, 95 % confidence interval=29.1–33.2 vs. non mutation carriers, 96.7, 95 % confidence interval=91.0–102.4; p<0.0001) (Figure 5B). ROC analysis indicated 51.4 μg/L (97.1 % sensitivity; 98.1 % specificity) and 58.2 μg/L (97.1 % sensitivity; 100.0 % specificity) to be the best values to single out mutation from non-mutation carriers, with an area under the curve of 0.9949 (95 % confidence interval=0.985–1, p<0.0001) (Figure 5C and D).

## Discussion

This study shows the analytical and clinical validation of a commercial progranulin ELISA kit for clinical detection of possible *GRN* mutation carriers. Several parameters were tested according to previously described procedures [32]. Among those, repeatability and intermediate precision resulted in CV’s lower than 5.6 and 10 %, respectively, decreasing even further when normalizing measured concentrations using an IC. The dilution factor was proven to range between 50- and 200-fold without any considerable disturbances to the outcome measure. However, sample dilution was adjusted from the manufacturer’s recommended 1:200 to 1:100, due to better analytical performance and for all samples to fall within the range of the calibration curve. This allows for analytical range extension and increased measurement accuracy. Spike recovery averaged at 96 % with even distribution indicating selective targeting for PRGN and minimal influence from other analytes.

Measurement range corresponded to those reported by the manufacturer, 6.25–400 μg/L, with values below the determined LLOQ considered unmeasurable, while above ULOQ needing to be further diluted. PRGN was also proved to withstand storage at several temperatures and to be resistant to freeze/thaw cycles. This way, samples endure up to three freeze/thaw cycles and room temperature for up to three days. Samples can be stored at 4 °C for up to 7 days, while waiting for the measurements. For longer periods of time, freezing is advised, as this protein is also stable for longer periods at −20 °C. Considering this assay measures plasma samples, the most common interference is hemolysis. This was tested as a comparison between non-hemolyzed and high degree of hemolysis samples from the same individuals. Although we did not assess interference in different degrees of hemolysis, interference at a high degree was deemed neglectable as it did not influence this protein level. Hook effect was considered minimal, because in this protocol, addition of sample and detection antibody are followed by two different rinsing steps, reducing the risk of excess antigen quenching the signal. Another common interference in this type of immunoassays are human anti-mouse antibodies (HAMA), however in this case, as assay diluent contains anti-HAMA, this was not tested. In general, this assay was proven to be a robust analytical tool to specifically assess PGRN concentrations in serum and plasma.

Clinical validation was tested by analyzing samples from individuals with different neurodegenerative diseases. Similarly to previous tested PGRN plasma assays, in this study, comparing PGRN levels between different neurodegenerative diseases show significant distinction from *GRN* mutation and non-*GRN* mutation carriers, without any differences from symptomatic to non-symptomatic *GRN* mutation carriers [20, 22]. As pre-symptomatic mutation carriers already show decreased levels of PGRN, we need to define a better age of onset to start administering PGRN enhancing therapies. Clinical validation of assays like the one evaluated in this paper can help provide a tool for earlier screening.

To use plasma progranulin as a diagnostic biomarker, one of the most important factors to consider is minimizing false negatives. This requires carefully determined cut-off points, so no potential mutation carriers are left out, being crucial if this method is to be included in progranulin-targeting therapeutic trials. As such, implementing this assay as a tool in clinical laboratory practice, with its potential to differentiate between *GRN* mutation carriers and non-mutation carriers, will allow only probable mutation carriers to be sent for genetic screening, thus reducing the need for genetic testing. The results of this study add up to the already established diagnostic value of this biomarker with a clinically validated assay with an optimal cutoff value of 57 μg/L, that provides high sensitivity and specificity in distinguishing *GRN* mutation carriers form non-*GRN* mutation carriers [20], [21], [22]. This threshold value was lower than the values reported previously. This could be due to the smaller study population, although still substantial for a rare genetic form of FTD. In future studies, a more extensive population can be used to better prove the establishment of this cut-off.

All confirmed *GRN* mutation samples except one were correctly classified with the established cutoff. This one incorrectly classified sample had a value of 57.5 μg/L. This event can be due to the presence of minor haplotypes, like rs1990622 in *TMEM106*B, that were previously proven to delay disease penetrance by upregulation of PGRN levels [25, 33, 34]. Similarly, two samples were below the cutoff on the non-mutation carrier group with 34.5 μg/L and 45.3 μg/L. Both patients belonged to the bvFTD group, as such, the reduced levels might be derived from minor risk factors like SNPs at the *PSAP* and *CDH23* locus, which have been proven to be associated with decreased PGRN levels [8].

Overall, with this clinical validation report, we demonstrate robust analytical and diagnostic performance of the commercial progranulin kit for implementation in clinical routine. This allows a straightforward screening of patients to identify individuals who are likely *GRN* mutation carriers. This assay might also be used as a primary outcome measure to test the effect of novel PGRN-targeting drugs and therapies.
